# Long-term outcomes of mitral valve repair in children

**DOI:** 10.3389/fcvm.2024.1454649

**Published:** 2024-10-01

**Authors:** Osman Nuri Tuncer, Serkan Ertugay, Mahsati Akhundova, Ertürk Levent, Yüksel Atay

**Affiliations:** ^1^ Department of Cardiovascular Surgery, Faculty of Medicine, Ege University, Izmir, Türkiye; ^2^ Department of Pediatric Cardiology, Faculty of Medicine, Ege University, Izmir, Türkiye

**Keywords:** mitral valve annuloplasty, cardiac surgical procedures, mitral regurgitation, congenital heart disease, mitral stenosis (MS)

## Abstract

**Background:**

Mitral Valve Disease in children presents unique challenges due to the wide range of associated pathologies and the complexities of pediatric cardiac anatomy. Mitral valve repair in this demographic is preferred over replacement due to the drawbacks associated with prosthetic valves in young patients, such as the need for long-term anticoagulation and issues with prosthetic size and growth.

**Methods:**

This retrospective study reviewed pediatric patients under 18 years who underwent mitral valve repair between January 2002 and December 2023. Exclusion criteria included patients with atrioventricular septal defects or single-ventricle physiology. Surgical outcomes were assessed using preoperative and postoperative transthoracic echocardiography, with follow-up data analyzed via Kaplan-Meier survival estimates.

**Results:**

The study included 47 patients with a median age of 4 years. Surgical techniques varied based on the specific mitral valve pathology. The overall early mortality was 6.3%, and the one and ten-year survival rates were 93.6 ± 3.6% and 91.4 ± 4.1%, respectively. Most patients showed improved or stable postoperative cardiac function during a median follow-up of 105 months. Notably, the rate of freedom from re-operation at ten years was 85.1 ± 6.9%, highlighting the durability of the surgical interventions.

**Conclusions:**

Mitral valve repair in children demonstrates favorable long-term outcomes with low mortality and reoperation rates, particularly when performed at an older age to accommodate growth and avoid the complexities of smaller, more delicate cardiac structures. These findings suggest that mitral valve repair should be considered a viable and effective option for managing pediatric MVD, with a personalized approach essential for optimizing outcomes.

## Introduction

Mitral valve disease (MVD) is less frequently observed in children compared to adults, but there can be a wider range of pathologies due to the coexistence of congenital heart defects (1). The small, immature, and fragile nature of mitral valve structures in the neonatal and infantile periods can make surgical treatment of MVD challenging in the pediatric population (2). Difficulties in obtaining appropriately sized prostheses, the necessity of long-term anticoagulant use, poor long-term outcomes, and a high risk of patient-prosthesis mismatch all contribute to making replacement surgery less favorable for pediatric patients. This adds further complexity and makes the surgical management of MVD more challenging (3, 4).

This article aimed to examine the outcomes of mitral valve repair in pediatric patients and determine the factors that contribute to success or complications. A multidisciplinary approach and the experience of specialist physicians play crucial roles in the treatment of pediatric patients with MVD. The treatment plan should be personalized according to the patient’s specific conditions and characteristics of the disease.

## Materials and methods

Between January 2002 and December 2023, a retrospective review was conducted of pediatric patients aged <18 years who underwent surgery for congenital or acquired mitral valve disease at our clinic. Patients with atrioventricular septal defects or single-ventricle physiology were excluded from the study. Data of the patients were obtained through a review of hospital and national medical records. Ethical approval for the study was obtained from the institution. (Approval number: 2024-2899 24-5T/33).

### Pathology and determination of MVD severity

Mitral valve function was evaluated preoperatively using transthoracic echocardiography (TTE). Mitral regurgitation (MR) was graded between I and IV based on abnormal jet flow into the left atrium. Mitral valve pathologies were roughly classified according to the Carpentier’s Functional Classification (5). Before cardiopulmonary bypass, the patients were re-evaluated in detail using intraoperative transesophageal echocardiography (TEE), and the morphology of the mitral valves was examined. During this examination, the presence of any other abnormalities was also assessed.

### Surgical indications and techniques

Unlike in adult patients, there are no definitive guidelines indicating the surgical indications for mitral valve surgery in pediatric patients (6). Therefore, the surgical indication was individually evaluated for each patient, and a decision for the operation was made by a multidisciplinary council involving a pediatric cardiologist, pediatric intensivist, and pediatric cardiac surgeon based on valve pathology, the presence of concomitant cardiac anomalies, and the patient’s clinical condition. Surgical procedures were preferably performed on patients at an advanced age to ensure a larger surgical field and as mature tissues as possible.

All patients underwent surgery under general anesthesia using cardiopulmonary bypass. In most cases, a median sternotomy was performed as the surgical incision; however, in recent cases that required no additional surgical intervention or only involved an atrial septal defect, right anterolateral thoracotomy was preferred. Intermittent blood cardioplegia was used for myocardial protection during these procedures. Mild hypothermia (28–32 degrees) was used in all patients except for one case. The exceptional case was a patient with both interrupted aortic arch pathology and mitral valve disease, and total circulatory arrest lasting 18 min was employed during arch reconstruction. Mitral valve exploration was performed either through a direct left atriotomy or by opening the interatrial septum.

MR due to annular dilatation and the absence of an appropriate ring size modified Kay-Whooler Annuloplasty using either teflon pledget or autologous pericardium was performed in these patients. An artificial ring was used for annular stabilization in all patients for whom a suitable ring was present.

The leaflet clefts were repaired using 5/0 prolene without causing distortion, extending to the most distal part as much as possible. In cases where regurgitation was near the commissures peripherally, the commissural apposition area was closed using separate prolene sutures.

Quadrengular/triangular resection was performed in the patients with leaflet prolapse. For patients with chordal elongation, chordal shortening was performed, and artificial chordal implantation techniques were performed as necessary for those with chordal rupture.

Commissurotomy was performed in cases of mitral stenosis (MS) due to commissural fusion, peeling in cases with tissue accumulation limiting leaflet movement, and papillary muscle division in cases of chordal restriction.

All patients were evaluated for mitral valve function by intraoperative TEE after cardiopulmonary bypass and TTE before discharge. TTE was performed at the first, third and sixth months of follow-up. Patients with no deterioration in mitral valve function were followed up with annual TTE.

### Statistical analysis

Continuous data were expressed as median (range). Analysis of overall survival and freedom from MV re-operation or MV replacement were performed with the Kaplan—Meier method. Jamovi for Mac version 2.5.2 was used for all statistical analysis.

## Results

47 patients < 18 years, were included in this study. The median age was 4 years (min 4 months max 17 years) and weight was 4 kg (range, 4–78 kg). Of the patients, 12.7% (*n* = 6) were under one year of age and 13 (27.6%) were under 10 kg. The proportion of female patients was 57.41% (*n* = 27). Among the patients, 63.8% (*n* = 30) had an additional cardiac pathology, and atrial septal defect was the most common congenital anomaly (27.6%). Some patients had more than one additional cardiac pathology. Down syndrome was the most frequent genetic syndrome in 12.8% (*n* = 6) of the patients. The demographic data of the patients are summarized in Table 1.

**Table 1:** Demographic data of patients.

Age		Median 4 year (3 month-17 year)	
Weight		Median 15 kg (4–78 kg)	
Gender			
Female		27 (%57,4)	
Male		20 (%42,6)	
Genetic/Metabolic Syndrome		8 (17.1%)	
Down Syndrome		6 (12.8)	
Williams Syndrome		4 (8.5%)	
MPS3		1 (2.1%)	
Shone		4 (8.5%)	
MR		33 (70.2%)	
2′		3 (6.4%)	
3′		19 (40.4%)	
4′		11 (24.8%)	
MS		14 (29.8%)	
10–15 mmHg		4 (8.5%)	
15–20 mmHg		6 (12.8%)	
>20 mmHg		4 (8.5%)	
Associated cardiac lesions		30 (63.8%)	
ASD		13 (27.7%)	
VSD		7 (14.9%)	
SADM		5 (10.6%)	
PDA		4 (8.5%)	
AoK		3 (6.4%)	
Absance of left pericard		1 (2.1%)	
IAA		1 (2.1%)	

Preoperative Functional Classification		Postoperative Functional Classification	
NHYA 1	4 (8.5%)	NHYA 1	36 (76.5%)
NHYA 2	15 (31.9%)	NHYA 2	8 (17.1%)
NHYA 3	24 (51.1%)		
NHYA 4	4(8.5%)		

MPS3, Mucopolysacchoridosis type3; MVI, Mitral regurgitation; MS, Mitral stenosis; ASD, Atrial septal defect; VSD, Ventricular septal defect; SADM, Subaortic discret membran; PDA, Patent ductus arteriosus; AoK, Aort coarctation, IAA, interrupted arotic arch When postoperative functional capacities were shown, patients with early mortality were excluded.

The most frequently observed pathology in patients was MR, which was determined at a rate of 70.2% (*n* = 33). Common reasons for MR are leaflet cleft and annular dilation. Papillary muscle/commissural fusion in 6 patients, accounting for 12.8%, was the most common cause of MS. Patients data related to mitral valve pathologies are detailed in Table 2 along with the Carpentier functional classification.

**Table 2:** Carpentier’s functional classification for mitral valve lesions.

Mitral valve regurgitation	33 (%70,2)
Type 1 (normal leaflet motin)	21 (%44,8)
Annular dilatation	8 (%17)
Cleft leaflet	11 (%23,5)
Leaflet defect	2 (%4,3)
Type 2 (leaflet prolapsus)	10 (%21,3)
Elongated chordae	8 (%17)
Rupture chordae	2 (%4,3)
Type 3 (restricted leaflet motion)	2 (%4,3)
Fused commisures	2 (%4,3)
Mitral valve stenosis	14 (%29,8)
Type A (normal papillary muscle)	14 (%29,8)
Supravalvar ring	5 (%10,6)
Thickened leaflets	3 (%6,4)
Papiller muscle/commissural fusion	6 (%12,8)

Leaflet repair was the most frequently performed surgical tecnique for mitral insufficiency at a rate of 46.8%. Mitral commissurotomy was the most commonly used surgical technique for MS, with a rate of 21.2% (*n* = 10). Surgical techniques were combined with 39 patients (83%) based on the existing pathology, with one of the described techniques being necessary. The surgical techniques that were used are listed in Table 3.

**Table 3:** Techniques used for repair of MV.

Mitral regürgitation	
Cleft repair	16 (34%)
Modified Kay-Wooler annuloplasty.	14 (29.8%)
Ring annuloplasty	9 (19.1%)
Leaflet augmentasyon	5 (10.6%)
Edge-to-edge repair	8 (17%)
Chordae shortening	4 (8.5%)
Artificial chordae	2 (4.3%)
Quadrangular/triangular resection	4 (8.5%)
Mitral stenosis	
Commissurotomy	10 (21.2%)
Supravalvuler ring resection	6 (12.7%)
Papillary muscle splitting	8 (17%)
Peeling	4 (8.5%)

All patients with additional intracardiac pathologies underwent concomitant correction. Among the additional pathologies without intracardiac lesions, only the patient with an interrupted aortic arch underwent concomitant surgery. Of the three patients with coarctation of the aorta, two underwent preoperative mitral valve repair and one underwent balloon angioplasty after discharge. Of the 3 patients with coarctation, 2 had additional ASD, and these patients underwent concomitant surgery for ASD even if they did not undergo concomitant intervention for coarctation. No procedure was performed in the patient with an absent left pericardium.

**Figure 1:**
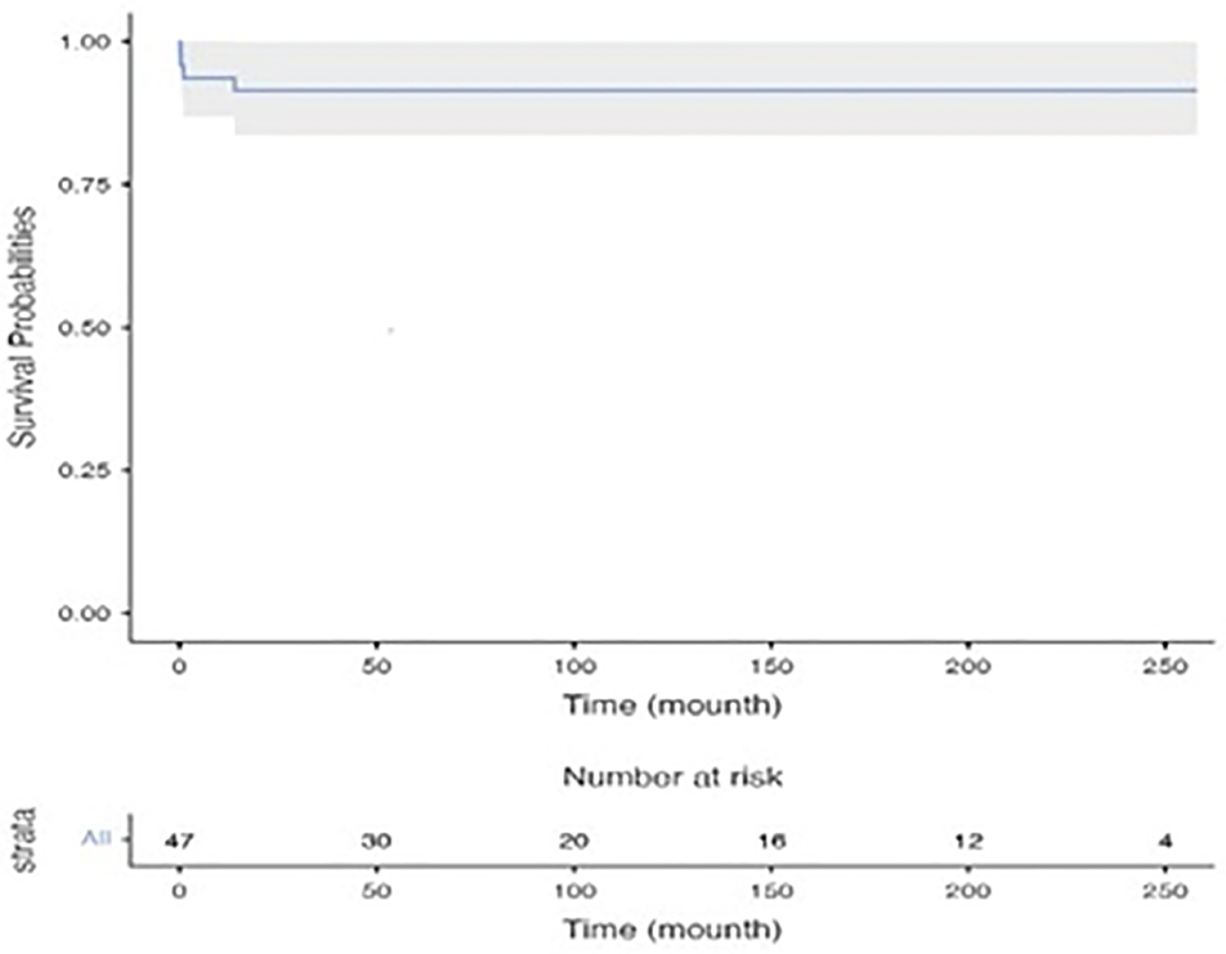
Kaplan-Meier curves showing overall survival.

Early mortality was observed in 3 patients (6.3%). Two of these patients were intubated and operated under inotrope support with poor functional capacity (NHYA IV). The cause of mortality in these two patients was considered to be low cardiac output syndrome and mortality occurred on the fourth and sixth post-operative days, respectively. The other patient had a prolonged hospital stay before the operation due to infective endocarditis and died 27th day after the operation due to sepsis. Late mortality was seen in only 1 patient (2%) and this patient was on the transplant list for non-compaction dilated cardiomyopathy. She died due to heart failure 8 months after mitral valve repair while she was on the waiting list for heart transplant. Only one patient under 1 year of age had mortality and the other 2 patients were over 1 year of age. The 1- and ten-year survival rates for patients were found to be 93.6 ± 3.6% and 91.4 ± 4.1%, respectively, as illustrated by the Kaplan-Meier curve depicting these data in Figure 1.

After the removal of patients with early mortality, the average follow-up period was 105 months (minimum, 6; maximum, 258). None of the patients were lost to follow-up. During follow-up, no worsening in functional capacity compared to pre-operation was observed in any patient; additionally, improvement in functional capacities was observed for all patients with low functional capacity who underwent surgery. Data on the pre- and postoperative functional capacities of the patients are provided in Table 1.

Before the operation, 11 patients had fourth-degree MR. After the operation, except for one patient, no other patient had a higher degree of MR than the first degree. The patient with postoperative grade 2 MR was under one year old and had a functional capacity of NHYA III. Considering the poor general condition of the patient and to avoid prolonging the cardiopulmonary bypass time further, intraoperative revision was not considered. The patient tolerated second-degree MR well during the postoperative period and was discharged on postoperative 11th day.

Patients who underwent surgery due to mitral stenosis were found to have a preoperative mean gradient of 17 mmHg (range 11–27 mmHg), while the postoperative mean gradient was 3.1 mmHg (range 2–4 mmHg).

No intraoperative reoperation was performed in any of the patients. Four patients underwent reoperation due to recurrent mitral valve pathology during the long-term follow-up, all of whom had previously undergone surgery for MR. None of our patients who underwent surgery for MS required reoperation. Three patients who underwent reoperation were under 1 year of age at the time of their initial repair. Two of these patients underwent repeat mitral repairs, and two underwent mitral valve replacement (MVR). One patient who underwent MVR for prosthetic valve dysfunction underwent repeat MVR five years later. In one patient who had undergone mitral repair, anterior leaflet indentation repair and ring annuloplasty were performed. After conducting a saline test on another patient who had undergone residual cleft repair and Modified Kay-Wooler annuloplasty, it was determined that there was a leakage issue between A1-P1. To rectify this situation, two stabilizing sutures were applied to the A1-P1 region. This patient was the patient was discharged with second-degree MR after the first operation and required a reoperation 5 years after the initial surgery.

**Figure 2:**
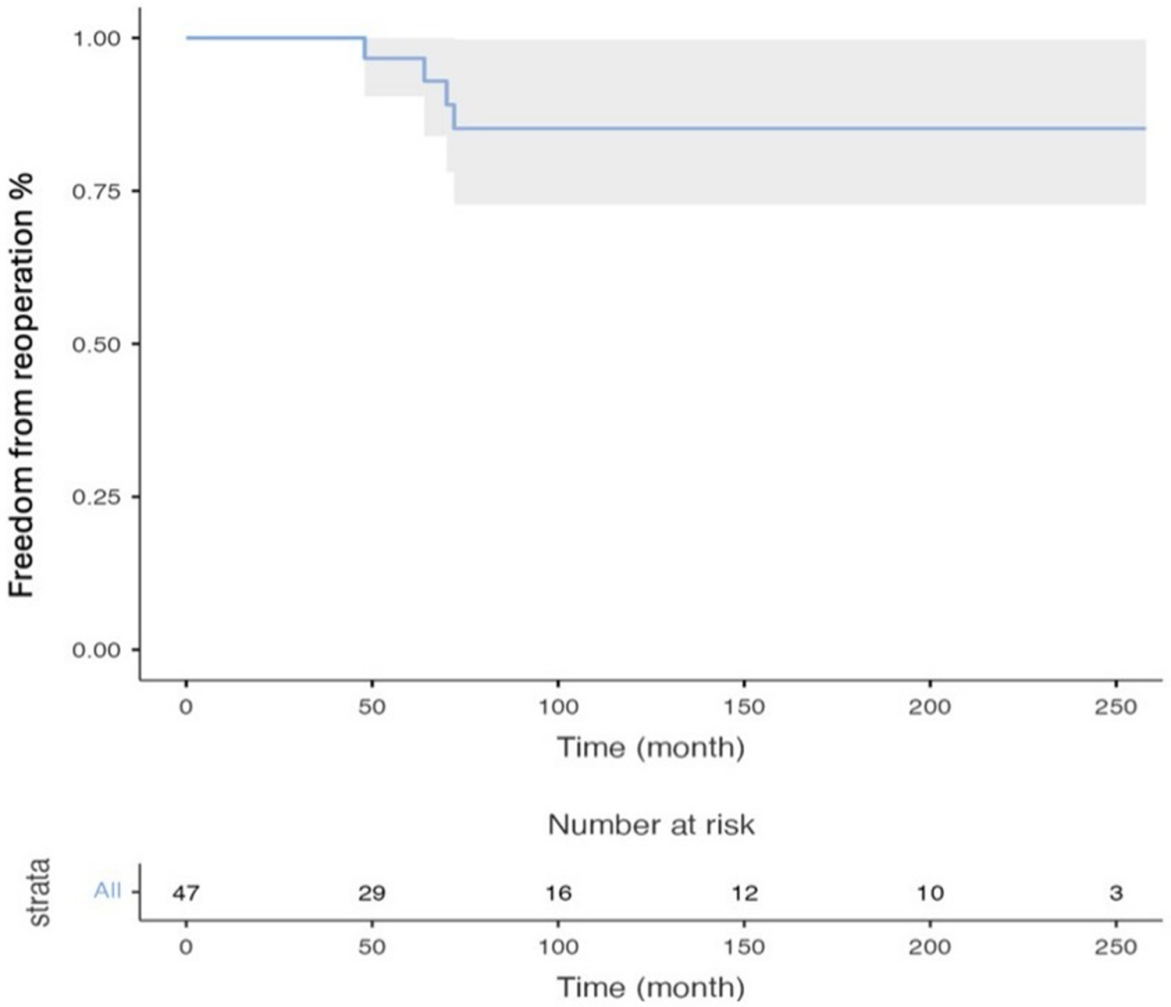
Kaplan-Meier curves showing freedom from re-operation due to MVD.

The rates of freedom from operation at 1,2,3 and10 years were calculated as 96.7 ± 3.3%, 92.9 ± 4.8%, 89.l ± 6% and 85.1 ± 6.9% respectively. The Kaplan Meier curve for freedom from operation is shown in Figure 2.

## Discussion

Mitral valve surgery in pediatric patients presents unique challenges due to their small and immature valve structures, a wide range of pathologies, and associated congenital anomalies. Prosthetic valve replacement is particularly challenging in this population due to limited prosthetic options, poor long-term outcomes, potential complications related to anticoagulant use, and incompatibility with patient growth (7, 8).

Surgical techniques for mitral valve reconstruction in pediatric patients have shown promising results. Despite the early mortality rate of 6.3% observed in our study, the 10-year survival rates and low reoperation rates at 1, 2, 3, and 10 years suggest the success of the procedures. Furthermore, throughout the average follow-up period of 105 months, none of the patients exhibited a decline in their functional capacity postoperatively. Moreover, those with low functional capacity prior to surgery demonstrated an improvement in their functional capacity. Our study shows that successful mitral valve repair in children can be performed in dedicated centers with low morbidity, mortality and reoperation rates.

In a study by Hetzer et al. reporting 20 years of experience in mitral repair on 111 patients under 18 years of age, MR was the most common mitral valve disease with a rate of 80.2%. The most common causes of MR were mitral leaflet cleft and annular dilatation (9). In other studies in the literature, MR constitutes the majority of the pediatric group operated for MVD. In our study, the most common causes of MR were determined as annular dilatation and cleft in accordance with the literature (10, 11).

The high rate of concomitant congenital anomalies in pediatric patients makes MVD pathology spectrum wide and repair challenging. In Hetzer’s study, 107 (96.3%) of the patients had additional congenital defects and 74 (66.7%) of them underwent concomitant surgery (9) and these data are compatible with our study. This situation shows that pediatric valve repair requires experience in congenital heart disease surgery. In our study, 28(59.5%) patients underwent concomitant surgery for additional pathologies.

Hetzer et al. achieved annular stabilization with the Modified Kay-Wooler technique in the majority of patients with annular dilatation and used an artificial ring in only 4 patients (3.6%) (9). Although the mean age of their patients was older than that of our study group, they did not prefer an artificial ring because they were concerned about the use of anticoagulants, even for a short period of time, subsequent somatic growth problems, and the risk of a particularly rigid prosthesis causing distortion of the cardiac cavities and/or contributing to left ventricular outflow tract obstruction. In our patient group, an artificial ring was used in all patients in whom a suitable sized ring for annular stabilization was available. Patients who received an artificial ring were treated with anticoagulant therapy for a short period of time (3 months), no anticoagulant-related complications occurred in any patient, and no left ventricular outflow tract stenosis requiring a procedure was detected in any patient. None of our patients who received artificial ring implantation required any re-intervention for cardiac reasons.Although there are studies emphasizing the development of severe MR in 25% of repairs performed without the use of a ring (12) it is also known that other annuloplasty techniques can be used successfully in children and prosthetic rings are not indispensable to achieve positive results (13). In our study, although there were a large number of patients who underwent annuloplasty without the use of a ring, the low long-term reoperation rate indicates that ring use is not mandatory.

Another consideration regarding ring usage is ring selection. In the literature, biodegradable rings are frequently encountered, especially in pediatric cases, due to their increased resistance to infections and their ability to be completely absorbed over time, thus not impeding annular growth (14, 15). Due to the difficulty in obtaining biodegradable rings, it was not possible to utilize them in our cases.

Even when only patients with annular dilatation are considered, it shows that there are no definitive surgical techniques for mitral repair. The patient’s age, functional capacity, presence of additional pathologies and the condition of the mitral valve structures are the main factors that determine the surgical technique. To adequately address the various pathologies that are commonly associated with pediatric MVD, it is essential to customize surgical methods based on each patient’s unique requirements. This personalized approach ensured the best possible outcome for these patients. In our study, we observed the use of several established surgical techniques in the treatment of patients, highlighting the complexity and variability of the cases. Some cases require a combination of multiple techniques to effectively address the intricate nature of the pathologies. This demonstrates the necessity of a comprehensive and adaptable approach when dealing with pediatric mitral valve disorders (16). The aim of a good repair should be a valve with satisfactory function rather than an ideal anatomical and morphologic valve. Because the main goal is to allow the patient to grow with a good functional capacity.

Early mortality was seen in 5 (4.5%) patients in the series of Hertzer et al. All of them had additional congenital anomalies and 4 patients were under 1 year of age. All early mortalities occurred in patients operated for MR. Late mortality was seen in 9 (8.1%) patients, only 1 of whom was operated for MS. In our study, only 1 patient under 1 year of age died in the early period, although the mean age was higher than our patients (median age was not specified in this study). In the study by Hezter et al. the number and proportion of patients under one year of age was higher than in our patient group (9). This supports our opinion that patients should be operated at the oldest possible age for better results.

In a study by Lee et al. involving 139 patients under 18 years of age, no early mortality was observed. In the late period, 3 (2.1%) patients who operated for MR died due to heart failure. Lee et al. reported an excellent 15-year survival of 97.1% (11). In a more recent study of 40 patients under 10 years of age by Mayr et al, operative mortality was seen in 2 (5%) and late mortality was seen in 4 (10%) patients. In Mayr’s series, the majority of patients with mortality were operated on for MR (10). In our case series, there were 3 (6.3%) early and 1 (2%) late mortality. In our series, mortality was not seen in any MS patient and all patients who died were patients who were operated for MR.

There are studies in the literature showing that mortality and morbidity are higher in patients operated for MS. In Hetzer’s series, the majority of patients with mortality were MS cases. In Lee’s and our case series, no mortality was seen in any patient operated for MS (9–11, 17).

Considering the long-term life expectancy of pediatric patients, the most important criterion for the success of mitral repair in this patient group should undoubtedly be the durability of the repair. While it is known that reoperation rates cannot be eliminated even in prosthetic valve replacement (18), reoperations after repair should be an acceptable situation.

In the series by Hetzer et al. a total of 15 (13.5%) patients required reoperation due to mitral valve pathology. Of these, 7 underwent re-repair and 8 underwent valve replacement. The overall freedom from reoperation rates were 86.2% at 5 years and 79.2% and 73.5% at 10 and 20 years, respectively. In Hertzer’s study, the risk factors for reoperation were determined as younger age, presence of additional intervention, duration of operation and valve function at discharge (9).

In the series of Lee et al. 9 (6.5%) patients required intraoperative revision and a total of 29 reoperations were performed to 26 (19.7%) patients. Eleven of these patients underwent 12 valve replacements. The majority of the patients requiring reoperation were patients who underwent Kay-Wooler annuloplasty during the initial operation due to MR. The 15-year freedom from operation rate was 76.9% (11). In the series of Mayr et al. reoperation was seen in 16 (40%) patients. Most of these patients (11 of 16) were patients who had been operated for MR. Of the 16 patients, 8 underwent re-repair and 8 underwent replacement. Two patients from each group required reoperation in the following period and these 4 patients underwent replacement. In this study, the rate of reoperation was higher in patients operated for MS, although not statistically significant (10).

In our series, 4 (8.5%) patients underwent re-operation, which is lower than the re-operation rates in the studies mentioned above. The reason for our low reoperation rates may be explained by the fact that we operated our patients at the oldest possible age. As patients grow older, their valve structures become more favorable for repair and a more durable repair may be possible. This may be the reason for the high mortality and reoperation rates in the series of Mayr et al. In the study of Mayr et al. the median age of the patients was 1.2 years and their weight was 8.2 kg (10).

## Conclusions

This study underscores the importance of individualized surgical assessment in pediatric patients with MVD, considering factors such as age, comorbidities, and surgical risk. Studies in the literature show that patients operated at a young age are more likely to be reoperated. For this reason, when patients who operated on at an older age as much as possible with a multidisciplinary follow-up as in our series, the possibility of reoperation will decrease.

There is a need for continued research and assessment of surgical outcomes in pediatric populations to further refine the surgical approaches for pediatric valve diseases. By doing so, we can continue to improve the long-term prognosis and quality of life of pediatric patients with mitral valve pathologies.

## Data Availability

The raw data supporting the conclusions of this article will be made available by the authors, without undue reservation.
